# Impact of meconium-stained amniotic fluid thickness on maternal infectious morbidity: a comprehensive clinical and microbiological analysis

**DOI:** 10.1007/s00404-024-07808-4

**Published:** 2024-11-07

**Authors:** Raneen Abu Shqara, Lior Lowenstein, Maya Frank Wolf

**Affiliations:** 1 Department of Obstetrics and Gynecology, Raya Strauss Wing of Obstetrics and Gynecology Galilee Medical Center, Nahariya, Israel; 2 Azrieli Faculty of Medicine, Bar Ilan University, Safed, Israel

**Keywords:** Meconium, Meconium aspiration syndrome, Chorioamnionitis, Endometritis, *Enterobacteriaceae spp.*

## Abstract

**Purpose:**

The aim of this study was to investigate the correlation between the thickness of meconium-stained amniotic fluid (MSAF) and maternal infectious morbidity.

**Methods:**

A retrospective study of 15,950 term singleton pregnancies at a tertiary hospital (2020–2024). Women were categorized into four groups based on the presence and thickness of MSAF: clear, light, intermediate, and thick. The co-primary outcomes were clinical chorioamnionitis and puerperal endometritis, defined as a composite maternal infectious morbidity. In women with intrapartum fever (IPF), chorioamniotic swabs were obtained and compared according MSAF thickness. Multivariate analysis identified predictors of a composite maternal infections and adverse neonatal outcomes.

**Results:**

Of the cohort, 13,745 had clear amniotic fluid, and 2,205 had MSAF (561 light, 1,426 intermediate, 218 thick). The incidence of maternal infections increased with MSAF thickness, with thick MSAF showing the highest rates of clinical chorioamnionitis (4.1%, *p* < 0.001) and endometritis (1.4%, *p* = 0.039). In IPF cases, thicker MSAF was associated with a higher prevalence of positive swab cultures, particularly of *Enterobacteriaceae* (61.9%). *Group B Streptococcus* (GBS) remained consistent across all MSAF groups. Multivariate analysis showed that MSAF levels were associated with increased maternal infectious morbidity (*p* < 0.001). Additional risk factors for maternal infections included nulliparity (*p* < 0.001), catheter balloon insertion (*p* = 0.004), prolonged ROM (*p* < 0.001), and cesarean delivery (*p* < 0.001). In contrast, only intermediate (*p* < 0.001) and thick MSAF (*p* < 0.001) correlated with adverse neonatal outcomes.

**Conclusion:**

Greater severity of MSAF is associated with increased maternal infectious morbidity, especially infections related to Enterobacteriaceae. Studies about preventive measures in cases of thick MSAF are warranted.

## What does this study add to the clinical work

**Table:** 

1. Thicker meconium-stained amniotic fluid was linked to higher risk of Enterobacteriaceae-related maternal infections. 2. Intermediate and thick but not light meconium-stained amniotic fluid correlated with adverse neonatal outcomes.	

## Introduction

Meconium-stained amniotic fluid (MSAF) is observed in 5% to 20% of laboring patients, with the incidence reaching approximately 27% in post-term pregnancies [1]. The presence of meconium in the amniotic fluid has been linked to fetal acidemia, hypoxia, and hypoxic-ischemic encephalopathy [2], meconium aspiration syndrome, seizures, and neonatal mortality [3–5].

The relationship between MSAF and clinical chorioamnionitis, as well as neonatal sepsis, has been documented, yet the precise mechanisms remain unclear [6]. Additionally, the correlation between prolonged labor and MSAF was described in the past [7].

Previous studies have explored the correlation between MSAF thickness with adverse maternal and neonatal outcomes. Some studies have shown that only thick MSAF is correlated with adverse perinatal outcomes [8, 9], while other studies showed no significant link between MSAF thickness and rates of infectious morbidity such as clinical chorioamnionitis [10, 11]. In women with prolonged rupture of membranes (ROM) and intrapartum fever (IPF), MSAF increased the risk of isolating *Enterobacteriaceae* from chorioamnionitic swabs, by 10.6-fold [12]. In a study examining the impact of MSAF on bacterial growth in amniotic fluid, it was observed that even low concentrations of meconium (up to 1.5 mg/ml) inhibited the growth of *Escherichia coli* [13]. In light of these conflicting findings, our study aimed to explore the relationship between the thickness of MSAF and the risk of maternal infectious morbidity. Additionally, we aimed to assess the distribution of pathogens in chorioamnionitic swab cultures relative to the varying degrees of MSAF thickness.

## Material and methods

### Study population and setting

This retrospective study included women hospitalized in a tertiary university-affiliated hospital between March 2020 and May 2024. The Institutional Review Board of our institution approved the study. Informed consent was not obtained due to the retrospective design. The data were extracted from our electronic medical records.

Included were women with a singleton pregnancy, at term (≥ 37 weeks), who underwent a trial of vaginal delivery with documented amniotic fluid color. Exclusion criteria included multiple pregnancy, major fetal anomalies, intrauterine fetal death, bloody amniotic fluid, elective cesarean delivery (CD), and missing data. The cohort was categorized into four groups based on the presence and degree of MSAF: clear, light, moderate, and thick. Demographic and background data were compared across groups. MSAF thickness was categorized by the clinical judgment of the midwife or obstetrician, and observations were systematically documented in the electronic medical records.

### Outcomes

The co-primary outcome were clinical chorioamnionitis and puerperal endometritis rates compared between women with clear amniotic fluid, light, intermediate and thick MSAF. Clinical chorioamnionitis was defined as maternal temperature greater than or equal to 39.0°C, or maternal temperature of 38.0–38.9°C and one additional clinical risk factor, such as maternal leukocytosis > 15,000/mm^2^, purulent cervical drainage and fetal tachycardia (> 160 beats/min) [14]. Clinical chorioamnionitis was treated by ampicillin 2 g four times a day and gentamicin 240 mg once daily [15, 16]. Puerperal endometritis was diagnosed based on body temperature ≥ 38°C in the absence of any other cause, together with an associated clinical finding such as uterine tenderness, purulent lochia, tachycardia, or abdominal pain. Secondary maternal outcomes comprised: CD, vacuum assisted delivery, manual removal of the placenta, intrapartum fever, postpartum hemorrhage, and hospitalization length in maternity wards. Secondary neonatal outcomes comprised: small for gestational age (defined as < 2500 g), macrosomia (defined as > 4000 g), Apgar 5 < 7, pH < 7.1, neonatal intensive care unit (NICU) admission, transient tachypnea of the newborn, asphyxia, respiratory distress syndrome, noninvasive and invasive ventilation, antibiotic administration in NICU, meconium aspiration syndrome, hypoglycemia, neonatal early onset sepsis and bacterial pneumonia.

Women who were known group B streptococcus (GBS) carriers were treated with intravenous ampicillin during labor. Women with prolonged ROM ≥ 18h received intrapartum ampicillin 2 gr q.i.d. A sub-analysis of clinical chorioamnionitis rates among women with MSAF (all levels) according to intrapartum prophylactic antibiotic administration was performed.

### Microbiological studies

According to our department protocol, in women with IPF, chorioamniotic swabs were obtained. After its extraction, the placenta was placed on a sterile surface, and the chorioamniotic space was separated with sterile gloves and sampled with sterile swab cultures [17]. The results of chorioamniotic membrane swabs and antimicrobial susceptibility tests were reviewed by an infectious disease specialist, and certain bacteria were defined as contaminants. The results of the included cultures were classified to four major groups of pathogens: gram-negative Enterobacteriaceae, GBS, anaerobes, *Enterococcus faecalis*. These were compared according to the presence and degree of MSAF (clear fluid, light, intermediate and thick MSAF).

#### Sample size calculation

To compare clinical chorioamnionitis rates between deliveries with clear amniotic fluid and thick MSAF, we used data from Tran et al.[18]. Reported rates were 2.3% for clear amniotic fluid and 5.9% for thick MSAF. To detect a 3.6% difference with 80% power and α = 0.05, the required sample size was calculated to be at least 3,629.

### Statistical analysis

Continuous variables are presented as medians and ranges, and qualitative variables as frequencies and percentages. Continuous variables were compared using the Kruskal–Wallis test, and categorical variables were analyzed using Pearson’s chi-squared test. A multivariate logistic regression model was used to assess maternal infectious morbidity (clinical chorioamnionitis and puerperal endometritis), controlling for possible confounders. Another model predicted adverse neonatal outcomes (comprised pH < 7.1, bacterial pneumonia, sepsis, invasive ventilation, meconium aspiration syndrome, respiratory distress syndrome, asphyxia), controlled for possible confounders. Statistical analysis was performed using IBM SPSS Statistics for Windows, version 27.0 (IBM Corp., Armonk, NY, USA).

## Results

A total of 15,950 women were included in the study, 2205 women MSAF, while the remaining 13,745 had clear amniotic fluid. Those with MSAF were categorized into three groups based on the severity of MSAF fluid: light MSAF (*n* = 561), intermediate MSAF (*n* = 1,426), and thick MSAF (*n* = 218). Maternal characteristics are detailed in Table 1. The mean maternal age across the groups ranged from 29.4 to 29.9 years, with no significant differences observed (*p* = 0.703). The number of pregnancies and deliveries also showed no significant differences among the groups. However, the thick MSAF group had the highest rate of nulliparity (45.4%, *p* < 0.001), while the rate of grand multiparity was similar. The prevalence of diabetes mellitus and hypertension did not differ significantly among the groups. However, the thick MSAF group had a higher mean gestational age (40.2 weeks) compared to the other groups (*p* < 0.001) and a higher rate of delivery beyond 41 weeks (*p* < 0.001). The duration of ROM (hours), prolonged ROM, GBS colonization status, and intrapartum antibiotic prophylaxis were similar between the groups. Additionally, the use of oxytocin for induction was more common in the thick MSAF group compared to the others (*p* < 0.001), while the rates of cervical ripening via catheter balloon or application of prostaglandin E2 inserts were similar.

**Table 1:** Patients’ characteristics and obstetrical history data

	Clear *N* = 13,745	Light MSAF *N* = 561	Intermediate MSAF *N* = 1426	Thick MSAF *N* = 218	*p*-value
Maternal age	29.6 (18–49.2)	29.9 (19.5–43.2)	29.8 (18–46.3)	29.4 (19–46)	0.703
Pregnancy number	2 (1–22)	2 (1–13)	2 (1–22)	2 (1–9)	0.065
Delivery number	2 (1–11)	2 (1–10)	2 (1–9)	2 (1–8)	0.076
Nulliparity	4642 (33.8)	200 (35.7)	531 (37.2)	99 (45.4)	< 0.001
Grand multiparity	666 (4.8)	27 (4.8)	70 (4.9)	12 (5.5)	0.976
Diabetes mellitus	1021 (7.4)	26 (4.6)	85 (6)	16 (7.3)	0.432
Hypertension	462 (3.4)	21 (3.7)	37 (2.6)	6 (2.8)	0.401
Previous CD	164 (1.2)	5 (0.9)	9 (0.6)	3 (1.4)	0.256
Pregnancy week	39.4 (37–42)	40 (37–42)	40 (37–42.4)	40.2 (37.2–42)	< 0.001
Delivery week ≥ 41	1286 (9.4)	81 (14.4)	218 (15.3)	40 (18.3)	< 0.001
Group B Streptococcal colonization	708 (5.2)	28 (4.9)	79 (5.5)	8 (3.7)	0.695
Prolonged ROM ≥ 18 h	741 (5.3)	26 (4.6)	68 (4.7)	12 (5.5)	0.680
ROM duration	1.9 (0–72)	2.4 (0–103)	2.3 (0–108)	1.7 (0–33.6)	0.235
Intrapartum antibiotic prophylaxis	1260 (9.2)	50 (8.9)	133 (9.3)	18 (8.2)	0.959
Catheter balloon insertion	1033 (7.5)	44 (7.8)	91 (6.4)	19 (8.7)	0.379
Prostaglandin E2 insertion	206 (1.5)	6 (1.1)	18 (1.3)	4 (1.8)	0.723
Induction by oxytocin	876 (6.8)	36 (6.4)	79 (5.5)	19 (8.3)	< 0.001

*MSAF* meconium-stained amniotic fluid, *CD* Cesarean delivery, *ROM* rupture of membranes,

Data are presented in *N* (%) or median (range)

### Obstetrical outcomes

The obstetrical outcomes are shown in Table 2. The incidence of IPF increased with the severity of MSAF, 2.2% in the clear amniotic fluid group, 6.6% in the light MSAF group, 6.8% in the intermediate MSAF group, and 9.6% in the thick MSAF group. (*p* < 0.001). Clinical chorioamnionitis rates similarly increased, rising from 0.8% in the clear amniotic fluid group to 1.1% in the light MSAF group, 1.6% in the intermediate MSAF group and up to 4.1% in the thick MSAF group *p* < 0.001. Puerperal endometritis rates similarly increased with MSAF thickness, from 0.3% in the clear MSAF group to 0.4%, 0.5%, and 1.4% in the light, intermediate, and thick MSAF groups, respectively (*p* < 0.001).

**Table 2:** Maternal outcomes according to presence and severity of the MSAF

	Clear *N* = 13,745	Light MSAF *N* = 561	Intermediate MSAF *N* = 1426	Thick MSAF *N* = 218	
CD	1720 (12.5)	83 (14.8)	213 (14.9)	47 (21.6)	< 0.001
CD due to non-reassuring fetal heart rate	551 (32.0%)	43 (51.8)	115 (54.0)	28 (59.6)	< 0.001
Vacuum assisted delivery	476 (3.5)	23 (4.1)	53 (3.7)	13 (6.0)	0.201
Manual removal of the placenta	359 (2.6)	16 (2.9)	40 (2.8)	6 (2.8)	0.960
Intrapartum fever	300 (2.2)	37 (6.6)	97 (6.8)	21 (9.6)	< 0.001
Clinical chorioamnionitis	105 (0.8)	6 (1.1)	23 (1.6)	9 (4.1)	< 0.001
Puerperal endometritis	42 (0.3)	2 (0.4)	7 (0.5)	3 (1.4)	0.039
Postpartum hemorrhage	452 (3.3)	20 (3.6)	55 (3.9)	6 (2.8)	0.656
Hospitalization length in maternity ward	2.1 (1–17)	2.1 (1–7)	2.1 (1–22)	2.2 (1–12.4)	0.003

*MSAF* meconium-stained amniotic fluid, *CD* Cesarean delivery

Data are presented in *N* (%) or median (range)

The rate of CD was highest in the thick MSAF group, 21.6%, compared to 14.9%, 14.8% and 12.5% in the intermediate MSAF, light MSAF, and clear amniotic fluid groups, respectively (*p* < 0.001). Similarly, the rate of CD due to non-reassuring fetal heart rate was highest in the thick MSAF group (59.6%), compared with other groups, (54.0%, 51.8% and 32.0%, respectively, *p* < 0.001). While the need for vacuum-assisted delivery, manual removal of the placenta and postpartum hemorrhage did not differ significantly among the groups. Additionally, the length of hospitalization in the maternity ward was slightly longer for women in the thick MSAF group (median 2.2 days) compared to other groups (*p* = 0.003).

In a sub-analysis of women with meconium-stained amniotic fluid (all levels), the rates of clinical chorioamnionitis were similar between those who received intrapartum antibiotic prophylaxis and those who did not: 5/201 (2.5%) vs. 33/2004 (1.6%), *p* = 0.382.

### Perinatal outcomes

The perinatal outcomes are shown in Table 3. The thick MSAF group had higher rates of Apgar score (< 7) at 5 min (0.9%) compared to those in the intermediate MSAF group (0.1%), light MSAF group (0.2%), and clear fluid group (0.2%), *p* < 0.001. The incidence of umbilical cord pH (< 7.1) also increased with MSAF thickness, from 1.4% in the clear fluid group to 0.9%, 1.4% and 6.9% in the light, intermediate and thick MSAF groups (*p* < 0.001) Meconium aspiration syndrome and NICU admission rates were significantly higher in the thick MSAF group, with rates of 4.6% and 12.4% respectively, compared to other groups (*p* < 0.001 for both). Furthermore, the thick MSAF group showed a higher prevalence of respiratory distress syndrome (2.3%, *p* < 0.001), transient tachypnea of the newborn (4.6%, *p* < 0.001), noninvasive ventilation (8.3%, *p* < 0.001), the need for antibiotic administration in the NICU (9.2%, *p* < 0.001), and hypoglycemia (0.9%, *p* = 0.015).

**Table 3:** Neonatal outcomes according to presence and severity of the MSAF

	Clear *N* = 13,745	Light MSAF *N* = 561	Intermediate MSAF *N* = 1426	Thick MSAF *N* = 218	*p*-value
Birthweight < 2500 g	385 (2.8)	9 (1.6)	27 (1.9)	5 (2.3)	0.079
Birthweight > 4000 g	768 (5.6)	42 (7.5)	120 (8.4)	20 (9.2)	< 0.001
Apgar 5 < 7	26 (0.2)	1 (0.2)	2 (0.1)	2 (0.9)	0.106
pH < 7.1	190 (1.4)	5 (0.9)	9 (0.6)	15 (6.9)	< 0.001
NICU admission	389 (2.8)	23 (4.1)	71 (5.0)	27 (12.4)	< 0.001
Transient tachypnea of the newborn	126 (0.9)	9 (1.6)	27 (1.9)	10 (4.6)	< 0.001
Asphyxia	8 (0.1)	1 (0.2)	1 (0.1)	2 (0.9)	< 0.001
Respiratory distress syndrome	15 (0.1)	2 (0.4)	1 (0.1)	5 (2.3)	< 0.001
Noninvasive ventilation	113 (0.8)	11 (2.0)	29 (2)	18 (8.3)	< 0.001
Invasive ventilation	21 (0.2)	0 (0)	4 (0.3)	3 (1.4)	< 0.001
Antibiotic administration in NICU	164 (1.2)	9 (1.6)	36 (2.5)	20 (9.2)	< 0.001
Meconium aspiration syndrome	7 (0.1)	3 (0.5)	11 (0.8)	10 (4.6)	< 0.001
Hypoglycemia	40 (0.3)	4 (0.7)	10 (0.7)	2 (0.9)	0.015
Sepsis	5 (0)	0 (0)	2 (0.1)	0 (0)	0.316
Bacterial pneumonia	7 (0.1)	1 (0.2)	2 (0.1)	1 (0.5)	0.058

*MSAF* meconium-stained amniotic fluid, *NICU* neonatal intensive care unit

Data are presented in *N* (%) or median (range)

### Multivariate analysis

Table 4 presents the results of multivariate logistic regression model for predicting maternal infectious morbidity. Nulliparity was strongly associated with an increased risk of maternal infections, with an odds ratio (OR) of 2.35 (95% confidence interval (CI) 1.91–2.88, *p* < 0.001). Similarly, catheter balloon insertion OR 1.53 (95% CI 1.15–2.03, *p* = 0.004) and CD OR 1.84 (95% CI 1.44–2.34, *p* < 0.001) were significant predictors. Increased ROM duration further increased the risk of maternal infections OR 1.9 (95% CI 1.48–2.45, *p* < 0.001). Light, intermediate, and thick MSAF were strong predictors of maternal infections. OR 2.57 (95% CI 1.76–3.75, *p* < 0.001) for light MSAF, OR 4.66 (95% CI 3.61–6.00, *p* < 0.001) for intermediate MSAF, and 3.91 (95% CI 2.38–6.41, *p* < 0.001) for thick MSAF. Conversely, factors such as pregnancies extending beyond 41 weeks, oxytocin induction, and epidural anesthesia did not show significant associations with maternal infections.

**Table 4:** The adjusted odds ratio estimates from a multivariable logistic regression model of maternal infectious outcome^a^

	Adjusted odds Ratio	95% confidence interval	*p*-value
Nulliparity	2.35	1.91–2.88	< .001
Pregnancy week ≥ 41	1.22	0.92–1.61	0.162
Catheter balloon insertion	1.53	1.15–2.03	0.004
Prostaglandin E2 insertion	1.01	0.48–2.13	0.988
Oxytocin induction	1.08	0.79–1.48	0.633
Epidural anesthesia	0.98	0.54–8.25	0.876
Cesarean delivery	1.84	1.44–2.34	< .001
ROM duration	1.9	1.48–2.45	< .001
Light MSAF	2.57	1.76–3.75	< .001
Intermediate MSAF	4.66	3.61–6	< .001
Thick MSAF	3.91	2.38–6.41	< .001

^a^A composite of clinical chorioamnionitis and puerperal endometritis

*MSAF* meconium-stained amniotic fluid, *ROM* rupture of membranes

Table 5 presents the results of multivariate logistic regression model for predicting adverse neonatal outcomes. Among the MSAF categories, intermediate MSAF OR 2.31 (95% CI 1.67–3.16, *p* < 0.001) and thick MSAF OR 6.16 (95% CI 3.88–9.80, *p* < 0.001) were significantly associated with adverse neonatal outcomes. IPF and ROM duration demonstrated an association with adverse perinatal outcomes, OR 5.39 (95% CI 3.48–8.34, *p* < 0.001), and OR 1.49 (95% CI 1.08–2.06, *p* = 0.015), respectively. CD was also associated with a higher rate of adverse neonatal outcomes, OR 1.82 (95% CI 1.39–2.38, *p* < 0.001). Birthweight categories (< 2500 g) and (> 4000 g) were also significant predictors of adverse neonatal outcomes, OR 4.15 (95% CI 2.80–6.15, *p* < 0.001), and OR 1.52 (95% CI 1.01–2.29, *p* = 0.046), respectively.

**Table 5:** The adjusted odds ratio estimates from a multivariable logistic regression model of adverse neonatal outcomes^a^

	Adjusted Odds Ratio	95% confidence interval	*p*-value
Nulliparity	0.93	0.83–1.03	0.167
Pregnancy week ≥ 41	0.76	0.51–1.14	0.184
Catheter balloon insertion	1.15	0.78–1.7	0.485
Prostaglandin E2 insertion	0.65	0.2–2.06	0.462
Oxytocin induction	1.13	0.76–1.66	0.553
Intrapartum fever	5.39	3.48–8.34	< .001
CD	1.82	1.39–2.38	< .001
Birthweight < 2500g	4.15	2.8–6.15	< .001
Birthweight > 4000g	1.52	1.01–2.29	0.046
ROM duration	1.49	1.08–2.06	0.015
Light MSAF	1.21	0.67–2.15	0.529
Intermediate MSAF	2.31	1.67–3.16	< .001
Thick MSAF	6.16	3.88–9.80	< .001

^a^A composite of: pH < 7, pneumonia, sepsis, invasive ventilation, MAS, RDS, asphyxia

*CD* Cesarean delivery, *ROM* rupture of membranes, *MSAF* meconium-stained amniotic fluid,

Data are presented in *N* (%) or median (range)

### Microbiological studies

**Fig. 1:**
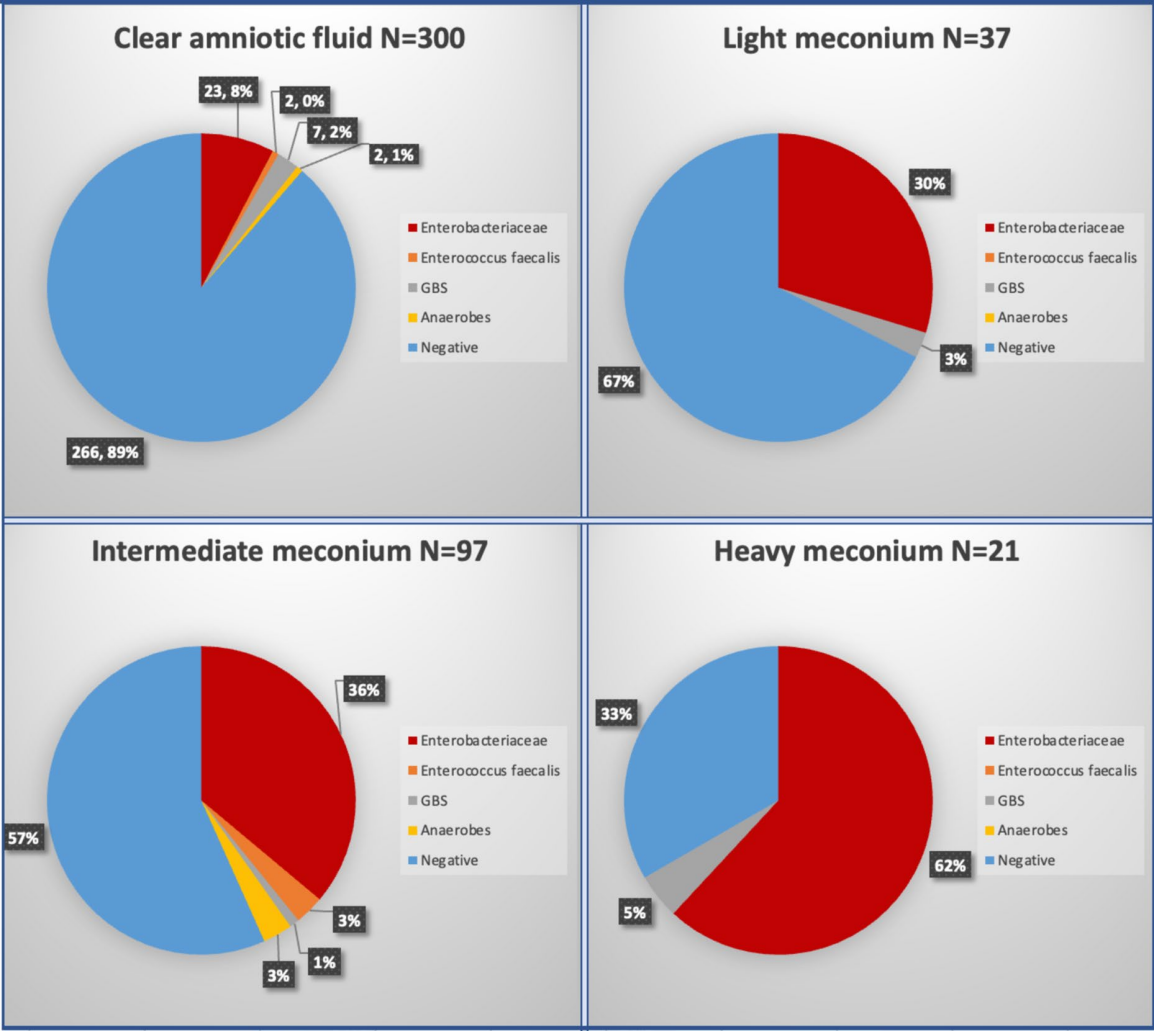
Pathogen distribution in chorioamniotic swabs in women with intrapartum fever, according to presence and severity of meconium thickness

Higher rates of positive chorioamniotic swab cultures were detected in those with thick MSAF (66.7%) than intermediate MSAF (43.2%), light MSAF (32.4%) and clear fluid (11.3%), *p* < 0.001. There was a significant correlation between the presence and thickness of MSAF and the isolation of Enterobacteriaceae in women with IPF. Notably, Enterobacteriaceae were detected in 61.9% of cases with thick MSAF, while intermediate, light MSAF, and clear fluid showed rates of 29.7%, 36.1%, and 7.7%, respectively (*p* < 0.001). No significant differences were observed for the presence of Enterococcus faecalis, GBS, or anaerobes across the different categories of MSAF (Fig. 1).

## Discussion

### Principal findings

The rates of clinical chorioamnionitis and puerperal endometritis were observed to increase proportionally with the severity of MSAF. In women with intrapartum fever, thicker MSAF was associated with a higher prevalence of *Enterobacteriaceae* in chorioamnionitic swabs, though GBS isolation rates remained consistent. Multivariate analysis demonstrated that all MSAF degrees were associated with an increased risk of maternal infectious morbidity. Additional risk factors for maternal infections identified in the analysis included nulliparity, catheter balloon insertion, prolonged ROM duration, and CD. In contrast, only intermediate and thick MSAF correlated with adverse neonatal outcomes, while light MSAF did not show the same association.

### Comment

#### Maternal morbidity

We found higher rates of clinical chorioamnionitis and puerperal endometritis in those with increasing thickness of amniotic fluid. The increased risk of clinical chorioamnionitis in women with thick MSAF is consistent with previous studies, which have shown that the incidence of clinical chorioamnionitis in cases of MSAF can rise significantly, from 2 to 8% [19]. Previous studies showed that MSAF enhances bacterial proliferation in amniotic fluid by serving as a nutrient-rich growth medium, while simultaneously inhibiting the fluid’s natural bacteriostatic properties and disrupting host defense mechanisms, such as phagocytosis [20]. On the other hand, it has also been theorized that the presence of inflammatory products in amniotic fluid may induce fetal bowel peristalsis when ingested by the fetus, potentially leading to the passage of MSAF [8, 21]. Studies indicate that patients with preterm labor and intact membranes who present with MSAF are more likely to have positive bacterial cultures compared to those with clear amniotic fluid (33% vs. 11%) [8]. Similarly, studies of term deliveries have shown that the presence of MSAF in cases of clinical chorioamnionitis is associated with a significantly higher rate of bacterial invasion—19.6% compared to 4.7% in cases without MSAF [21]. In our cohort of women with IPF, the rate of positive chorioamnionitic swabs varied between 32.4% and 66.7% among these with meconium-stained amniotic fluid, depending on the thickness of the MSAF. The highest rates were seen with thick MSAF, while the positive cultures rates in cases with clear fluid was only 11.3%.

Interestingly, while earlier studies have suggested that amniotic fluid is generally a poor culture medium for bacteria like *Escherichia coli *[22, 23], our findings, together with previous studies, indicate that the presence of sufficient MSAF can transform amniotic fluid into an excellent growth medium for Enterobacteriacae [22–24]. Other pathogens such as GBS, enterococcus and anaerobes appeared unaffected by MSAF thickness. Paradoxically, in a previous study, even the smallest concentration of meconium (1 mg/ml) led to a significant increase in GBS growth in MSAF [13]. The discrepancy between our findings and those of other studies likely stems from differences in methodology. While previous research used amniotic fluid cultures, our study relied on chorioamnionitic swab cultures, where contamination cannot be entirely ruled out.

The higher rate of CD associated with thick MSAF aligns with previous findings [25]. However, unlike the study by Gluck O et al., we found higher rates of CD due to non-reassuring fetal heart rate [10]. This is supported by the correlation between MSAF and fetal distress [26]. A study by Schreiber H et al., showed that women experiencing IPF and MSAF during labor face an elevated risk of CD compared to those with IPF alone [27]. Interestingly, a previous meta-analysis highlighted that while amnioinfusion significantly lowered cesarean delivery rates related to fetal distress, it did not show a corresponding reduction in the incidence of puerperal endometritis [28].

#### Neonatal adverse outcome

The correlation between MSAF thickness and perinatal outcomes remains a subject of debate. For example, some studies did not show correlation between MSAF thickness and perinatal morbidity [29, 30], while others found a strong correlation [31, 32]. We found that only intermediate and thick MSAF were associated with adverse neonatal outcome, this is similar to the findings of a previous study by Gluck O et al., [10]. While previous studies focused mainly on Apgar score, NICU admission, meconium aspiration syndrome, and advanced resuscitation, our larger sample enabled us to examine additional outcomes, for example, we found an association between MSAF thickness and antibiotic administration in NICU, respiratory distress syndrome, transient tachypnea of the newborn, invasive and non-invasive ventilation support, and hypoglycemia. Interestingly, a previous Cochrane showed that neonatal prophylactic antibiotic administration in cases of MSAF did not reduce the rate of neonatal infections [33].

While our study did not find a direct correlation between MSAF thickness and neonatal early-onset sepsis, previous studies have shown an increased rate of bacterial vertical transmission to the newborn in the presence of MSAF [34]. Additionally, the rates of meconium aspiration syndrome were observed to increase progressively with the thickness of MSAF. Notably, a Cochrane review demonstrated that amnioinfusion significantly reduces the risk of meconium aspiration syndrome (OR 0.33, 95% CI 0.22–0.51) [28].

Interestingly, fetal exposure to MSAF during labor was associated with a reduced risk of long-term respiratory-related hospitalizations and infectious morbidity in the offspring, possibly due to alterations in the offspring’s microbiome [35, 36].

### Clinical and research implications

The observed positive correlation between MSAF thickness and maternal infectious outcome prompt targeted studies about effective preventive strategies. Notably, a Cochrane review [22] of two randomized controlled study involving 362 participants demonstrated that administering ampicillin-sulbactam versus saline to women with meconium-stained amniotic fluid reduced the incidence of clinical chorioamnionitis. However, this intervention did not significantly affect rates of neonatal early-onset sepsis, NICU admissions, or puerperal endometritis. Enterobacteriaceae was identified as the leading pathogen in women with IPF and MSAF. The limited efficacy of ampicillin-sulbactam in preventing adverse outcomes may be attributed to the increasing resistance of Enterobacteriaceae, with resistance rates reported to be between 20 and 80% [37]. These findings suggest that further research should explore the utility of broad-spectrum antibiotic administration, in women with thick MSAF, as this group showed the highest levels of infectious morbidity.

### Strengths and limitations

One of the primary strengths of this study is its relatively large sample size, including 2,205 women with MSAF, which exceeds the numbers reported in previous studies [29, 31, 38, 39]. Additionally, the inclusion of chorioamniotic swabs offers a detailed analysis of microbial presence and its association with meconium aspiration. However, several limitations must be considered. Firstly, the assessment of MSAF fluid thickness by the obstetrician or midwife during labor was subjective. Secondly, the retrospective nature of the study is a limitation, as it precluded the examination of the effects of amnioinfusion or antibiotic administration during labor. Interestingly, in a recent meta-analysis, meconium aspiration syndrome was shown to be reduced in those who received amnioinfusion [28]. Lastly, we did not report long term outcomes such as cerebral palsy and intellectual abilities.

## Conclusion

Increasing severity of MSAF was associated with maternal infectious morbidity and particularly Enterobacteriaceae-related infections. This underlines the importance of close monitoring and the potential need for preventive measures in cases where thick MSAF is present. Studies about preventive measures in cases of thick MSAF are warranted.

## Data Availability

The data that support the findings of this study are available on request from the corresponding author, [RAS]. The data are not publicly available due to ethical restrictions.
